# Premature Puberty and Thimerosal-Containing Hepatitis B Vaccination: A Case-Control Study in the Vaccine Safety Datalink

**DOI:** 10.3390/toxics6040067

**Published:** 2018-11-15

**Authors:** David A. Geier, Janet K. Kern, Mark R. Geier

**Affiliations:** 1 Institute of Chronic Illnesses, Inc., 14 Redgate Court, Silver Spring, MD 20905, USA; davidallengeier@comcast.net (D.A.G.); mgeier@comcast.net (M.R.G.); 2 CoMeD, Inc., 14 Redgate Court, Silver Spring, MD 20905, USA; 3 CONEM US Autism Research Group, 408 N. Allen Dr., Allen, TX 75013, USA

**Keywords:** ethylmercury, mercury, merthiolate, premature puberty, thiomersal

## Abstract

Studies suggest a relationship between exposure to endocrine disrupters, such as mercury (Hg), and premature puberty. Hg exposure from Thimerosal-containing hepatitis B vaccine, administered at specific intervals within the first six months of life, and the child’s long-term risk of being diagnosed with premature puberty (ICD-9 code: 259.1), was retrospectively examined, using a hypothesis-testing, longitudinal case-control design on prospectively collected data, in the Vaccine Safety Datalink (VSD). Cases diagnosed with premature puberty were significantly more likely to have received increased exposure to Hg from hepatitis B vaccines preserved with Thimerosal given in the first month after birth (odds ratio (OR) = 1.803), first two months after birth (OR = 1.768), and first six months after birth (OR = 2.0955), compared to control subjects. When the data were separated by gender, the effects remained among females but not males. Female cases, as compared to female controls, were significantly more likely in a dose-dependent manner to have received a greater exposure to Hg from hepatitis B vaccines preserved with Thimerosal, given in the first six months after birth (OR = 1.0281 per µg Hg). The results of this study show a dose-dependent association between increasing organic Hg exposure from Thimerosal-containing hepatitis B vaccines administered within the first six months of life and the long-term risk of the child being diagnosed with premature puberty.

## 1. Introduction

During puberty, a child will transition into adulthood, hormonally, physically, and psychologically [1]. With puberty, an individual will attain reproductive function and will undergo an accelerated linear growth rate. Premature or precocious puberty is when puberty occurs at an unusually early age. The classical criteria for premature puberty in girls are when she develops secondary sexual characteristics before eight years of age or has her first occurrence of menstruation before nine years of age. In boys, it involves the development of secondary sexual characteristics before nine years of age [1].

There are many complex factors, such as genetic, metabolic, or environmental, that can cause activation of the hypothalamic pituitary gonadal (HPG) axis and result in an early onset of puberty [1]. Mercury (Hg) is an established endocrine disruptor [2], and it was previously hypothesized that the interaction between HPG axis hormones and Hg exposure may increase the risk for a child entering premature puberty [3]. It was even suggested by investigators from the United States (US) Environmental Protection Agency (EPA) that it was of significant public health importance to include endocrine endpoints in the epidemiological study of human populations exposed to Hg [4].

Thimerosal is a Hg-containing compound (49.55% Hg by weight) added to some vaccines, as a preservative. It was present in most childhood vaccines, until 1999, when the American Academy of Pediatrics and US Public Health Service called for its removal from all vaccines, as soon as possible [5]. At the time of the recommendation for removal, some children were receiving about 237.5 micrograms (µg) of Hg, by two years of age. This amount was much higher than any amount given to infants in the past. This amount was estimated to be a major source of Hg exposure (about 50%) that some infants received, with breast milk being the other major source of Hg exposure [6]. 

Even though Thimerosal was reduced or eliminated from some vaccines in the US and other wealthy countries, in the early 2000s, it is still present in many vaccines in developing countries [7]. Furthermore, in the US, Thimerosal is still present in the tetanus toxoid vaccine, one of the meningococcal vaccines, and most of the influenza vaccines, each containing about 25 µg Hg [7]. The continued presence of Thimerosal in influenza vaccines is of particular concern because in 2003–2004, three doses of influenza vaccine were for the first time routinely recommended for administration to children, during the first 18–24 months of life. In addition, it is routinely recommended that all US children receive an annual influenza vaccine, every year [8]. Furthermore, pregnant women in the US are recommended to be given an influenza vaccine [8], and in developing countries, pregnant women are to receive both the influenza vaccine and the tetanus toxoid vaccine [9]. So, fetuses, infants, and children are still being exposed to Hg from Thimerosal.

A previous ecological study examined the association between premature puberty and exposure to Hg from Thimerosal-containing vaccines, using the computerized medical records within the Vaccine Safety Datalink (VSD). That study revealed a significant dose-dependent association between the average birth cohort doses of Hg received from Thimerosal-containing vaccines and the birth cohort rate of medically diagnosed premature puberty [10]. The purpose of this study was to attempt to confirm and extend this previous epidemiological study. The aim of this study was to longitudinally follow individual persons (i.e., not entire birth cohorts) in the VSD database. It was hypothesized that increasing Hg exposure from hepatitis B vaccines preserved with Thimerosal, which are injected within the first six months after birth, would significantly increase the long-term risk of a person receiving a premature puberty diagnosis.

## 2. Materials and Methods

The study was a hypothesis-testing, longitudinal case-control design on prospectively collected data that was retrospectively examined. The study protocol was approved by the US Centers for Disease Control and Prevention (CDC), the Institutional Review Board (IRB) of Kaiser Permanente North-West, and the IRB of Kaiser Permanente Northern California (approval date 5/1/2014; project identification code CR00003272). The data were analyzed at the secure Research Data Center of the National Center for Health Statistics in Hyattsville, MD, USA. The views expressed in this study do not necessarily reflect those of the CDC or those of Kaiser Permanente.

The VSD project was created in 1991 by the National Immunization Program (NIP) of the CDC. The VSD’s data collection and study methods have been previously described [11,12,13]. The project links medical event information, specific vaccine history, and selected demographic information from the computerized databases of several health maintenance organizations (HMOs). The data examined was collected between 1991 and 2000.

### 2.1. Determining the Population at Risk

A cohort of more than a million infants, enrolled in the VSD project, from Kaiser Permanente North-West, Kaiser Permanente Colorado, and Kaiser Permanente Northern California was analyzed using the Statistical Analysis System (SAS)^®^ analytics software (version 9.1 SAS^®^ software; SAS Institute Incorporated, located at 100 SAS Campus Dr., Cary, NC, USA). The CDC data provided to independent researchers was updated and available, until the end of the year 2000. The cohort examined was limited to accessible records for HMO-enrolled persons that were continuously enrolled from their date of birth and who also had records indicating gender.

### 2.2. Determining the Cases

In this study, the International Classification of Disease 9th revision (ICD-9) code 259.1 for premature puberty was used. To find the first occurrence of the diagnosis code of 259.1, the outcome files from this population were reviewed, including both inpatient and outpatient diagnoses. Only the first occurrence was counted in the situation where multiple occurrences of the same diagnosis in a child were found. Cases examined in this study included, both those vaccinated or not vaccinated. Further, the diagnosis of premature puberty had to follow the vaccines examined in this study in order to be sure that premature puberty was not diagnosed before vaccination

A total of four hundred and eighty-six cases diagnosed with premature puberty (females = 458, males = 28, female/male ratio = 16.36), born from 1991 to 2000, were detected. The children with the appropriate diagnosis code of premature puberty were then analyzed to establish the mean age of their initial premature puberty diagnosis, which was 2.94 years of age, and the standard deviation (SD) of the mean age of initial premature puberty diagnosis, which was 2.65 years of age.

### 2.3. Determining the Controls

To determine control subjects within the constraints of this database, control subjects who did not have a premature puberty diagnosis and who had a negligible chance of ever receiving a premature puberty diagnosis were identified. This was done to diminish the possibility of a misclassification of any control subjects. For control subjects to be selected they had to be enrolled from their birth, continuously, for a minimum of 5.59 years, which was the mean age of initial premature puberty diagnosis, plus the SD of the mean age of initial premature puberty diagnosis. Applying this criterion, the study identified 54,199 control subjects who did not have a premature puberty diagnosis (females = 26,209, males = 27,989, female/male ratio = 0.94) born from 1991 to 1995.

### 2.4. Hepatitis B Vaccine Exposure

The vaccine file for cases and controls was then reviewed to determine the exact dates of hepatitis B vaccine administration. The VSD that was made accessible to researchers (provided by the CDC) allowed researchers to study only one vaccine type per analysis. For example, a joint study of the combined exposure from the diphtheria, pertussis, and tetanus (DPT) vaccine and the hepatitis B vaccine, was not possible. However, it was possible to study hepatitis B combination vaccines, such as hepatitis B-haemophilus influenza type b (Hib). In addition, the data provided the ability to examine those cases and control subjects who were not given any hepatitis B vaccine doses. Hg exposure was assigned as 12.5 µg Hg per dose, for those receiving a pediatric hepatitis B vaccine or 0 µg Hg per dose, for those receiving either a combined hepatitis B-Hib (a Thimerosal-free hepatitis B vaccine) or neither of the aforementioned vaccines. Among the cases and controls, Hg from Thimerosal-containing hepatitis B vaccines ranged from a minimum exposure of 0 µg Hg to a maximum exposure of 37.5 µg Hg, administered within the first six months of life. These exposures to Hg were assigned irrespective of the manufacturer of the hepatitis B-containing vaccine examined.

### 2.5. Statistical Analyses

The Fisher’s exact test, contained in SAS, was utilized for statistical analyses to compare exposures to organic Hg from hepatitis B vaccines preserved with Thimerosal, which were injected in the first month after birth, in the first two months after birth and in the first six months after birth, among cases with a premature puberty diagnosis, compared to the control subjects (Groups I–III). The data were then analyzed in the same fashion except the cases and the control subjects were divided by gender so that males were compared to males (cases vs. control subjects) (Groups IV–VI) and female cases were compared to female controls (VII–IX). The logistic regression statistical test contained in StatsDirect (version 3.0.152; StatsDirect Ltd., 9 Bonville Chase, Altrincham, Cheshire WA14 4QA, UK) was utilized to examine the potential dose-dependent relationship between Hg exposures from hepatitis B vaccines preserved with Thimerosal, which were injected within the first six months after birth, among female cases in comparison to the female controls. In all statistical analyses those who received increasing doses of Hg from Thimerosal hepatitis B vaccines were compared to a 0 µg Hg reference group.

In this study, a two-sided *p* < 0.05 was designated as being statistically significant. The null hypothesis for each of the case-control comparisons was that there would be no difference in exposure to Hg from Thimerosal-containing hepatitis B vaccines among the cases, in comparison to the controls.

## 3. Results

Table 1 displays the relationship between exposures to Hg from Thimerosal-containing hepatitis B vaccines, administered at specific intervals within the first six months of life, among cases diagnosed with premature puberty, in comparison to controls. The associations ranged from the lowest (odds ratio = 1.768, *p* < 0.00001) in the cases with a premature puberty diagnosis in comparison to controls, when comparing those receiving 25 µg Hg from two doses of Thimerosal-containing hepatitis B vaccine in comparison to those receiving 0 µg Hg within the first two months of life, to the largest (odds ratio = 2.0955, *p* < 0.05) among the cases diagnosed with premature puberty in comparison to controls, when comparing those receiving 37.5 µg Hg in comparison to those receiving 0 µg Hg within the first six months of life.

Table 2 and Table 3 examine exposure to Hg from Thimerosal-containing hepatitis B vaccines, administered at specific intervals within the initial six months after birth, among male cases with a premature puberty diagnosis, compared to male control subjects, and female cases with a premature puberty diagnosis, compared to female control subjects, respectively. Table 2 shows that male cases diagnosed with premature puberty, in comparison male controls, were statistically no more likely to have received increased doses of Hg from Thimerosal-containing hepatitis B vaccines, within the first month of life, the first two months of life, or the first six months of life. By contrast, Table 3 reveals that female cases diagnosed with premature puberty, in comparison to female controls received significantly increased Hg exposure, within the first month of life (odds ratio = 1.865, *p* < 0.00001), the first two months of life (odds ratio = 1.838, *p* < 0.00001), and the first six months of life (odds ratio = 2.127, *p* < 0.05).

Table 4 shows the dose-dependent relationship between the increasing doses of Hg from hepatitis B vaccines preserved with Thimerosal, which were injected within the first six months after birth, among female cases with a premature puberty diagnosis, compared to the female controls. Overall, based on logistic-regression analysis, female cases with a premature puberty diagnosis were, statistically, significantly more likely than the female control subjects to have received higher doses of organic Hg from the hepatitis B vaccines preserved with Thimerosal, which were injected within the first six months after birth (odds ratio = 1.0281 per µg Hg, *p* < 0.005).

## 4. Discussion

The results observed in this study support the hypothesis that the risk of a premature puberty diagnosis, during the 1990s in the US, was due, at least in part, to the Hg exposure from Thimerosal-containing hepatitis B vaccine administered within the first six months of life. It was also found that the Hg exposure from Thimerosal-containing hepatitis B vaccines and the risk of being diagnosed with premature puberty occurred primarily in females where both the timing of administration and dose-dependence were important to modifying the effects observed.

It is important to consider that while this study observed significant effects mostly in females, it is possible that this study was statistically underpowered to find a significant relationship between Hg exposure from Thimerosal-containing hepatitis B vaccines and premature puberty in males because too few male cases were identified. It is generally accepted that females have more diagnosed premature puberty than males, but it is possible that many of the signs/symptoms of premature puberty are more subtle in males, and thus, there is under-diagnosis of premature puberty among males. Future studies should attempt to identify more male cases of premature puberty, so as to further discern the potential relationship between Hg exposure and premature puberty in males.

In further considering the results observed in this study, in the broader context of premature puberty trends in the US, it was previously reported that there was a significant advancement in the timing of puberty in the US, during the 1990s [14]. Ethnic, geographical, and socioeconomic backgrounds were described as providing equivocal explanations for the earlier onset of puberty, seen in the US, during the 1990s and, instead, it was suggested that endocrine-disrupting chemicals from the environment may be significantly influencing the timing of puberty [14]. The role of the endocrine disruptors from environmental pollution, on premature puberty, has received increased attention in recent years [15].

The results observed in this study are biologically plausible. It was previously described by investigators from the US EPA that Hg accumulates in the endocrine system and Hg is directly cytotoxic to the endocrine tissues. It was also described that Hg can bring about alterations in hormone levels, can influence sex hormones, and can up-regulate or down-regulate enzymes, within the steroidogenesis pathway [4].

Furthermore, recent research showed that intermittent neonatal administration of Thimerosal to young (neonatal) mice induced persistent significant dysregulation of the endocrine system in adult mice [16]. It was observed that there were significant elevations in the brain gene transcript levels of the gonadotropin-releasing hormone receptor (GNRHR), luteinizing hormone (LH), chorionic gonadotropin subunit alpha (CGA), prolactin (PRL), and follicle stimulating hormone beta (FSHB). It was also observed that there were significant elevations in the serum levels of PRL, FSH, and LH. These results are important because the GNRHR gene codes for the receptor that elicits the action of the gonadotropin releasing hormone (GnRH), to induce the production and release of the FSH and LH, from the pituitary. The alpha subunit of anterior pituitary secreting hormones, including the chorionic gonadotropin (CG), LH, FSH, and the thyroid-stimulating hormone (TSH), is encoded by the CGA gene, and it is the beta subunit of these hormones that dictates their respective receptor specificity and biological activity. Follicle growth/maturation and follicle luteinizing in female ovaries are mediated by the FSH and the LH pulses. Stimulation of mammary development, lactation, and reproduction are all mediated by prolactin, which is encoded by the PRL gene. Li et al. [16] concluded that long-lasting dysregulation of the HPG axis, in adults, was significantly induced by early intermittent neonatal exposure to Thimerosal.

The results of this study are also consistent with the previously mentioned ecological cohort study of the VSD database examining the potential relationship between Hg exposure from Thimerosal-containing infant vaccines and the risk of a child being diagnosed with premature puberty [10]. It was observed, in both studies, that the risk of a premature puberty diagnosis was increased in a similar order-of-magnitude dose-dependent fashion, by increasing exposure to Hg from Thimerosal-containing vaccines. The present study is differentiated from the previous VSD study because this study employed a longitudinal case-control methodology. As a consequence, in this study, individual persons were prospectively followed from birth, for specified periods of time, to ensure the capture of exposure to Thimerosal-containing hepatitis B vaccines and diagnostic status. This study also revealed, for the first time, that the endocrine disrupting effects of Hg exposure from Thimerosal-containing vaccines on the risk of a premature puberty diagnosis were more apparent in females than males. In addition, the results observed in this study are also consistent with the previously reported endocrine disrupting effects of Hg exposure, in some cases of the pink disease (this condition was frequently associated with the use of Hg-chloride in infants) [17]. It was described by investigators that some survivors of the pink disease were observed to have significant changes in adrenocortical sections and in androgen hormones, as well as exhibit clinical presentations of pseudohermaphroditism [17].

## 5. Strengths and Limitations

The overall strength of this study stems from the fact the medical records examined were prospectively collected, in their customary way, from continuous HMO-enrolled individuals. As such, many potential study biases, such as recall bias, were minimized because the healthcare providers, in making their diagnoses of the children examined in this study, did not have to associate the outcome of premature puberty with exposure to Thimerosal-containing hepatitis B vaccine. Further, neither the healthcare providers nor the children examined were aware of the eventual study design employed in this study. This study also attempted to minimize factors associated with healthcare-seeking activities or enrollment because all cases were enrolled from birth, until their premature puberty diagnosis, and all controls were enrolled from birth until 5.59 years-old.

Another strength of this study is that differences in exposure to Hg from Thimerosal-containing hepatitis B vaccines were, at least partially, the result of allowable differences in the timing for the routine vaccine schedule, and not merely the consequence of a few individuals receiving anomalous exposures to Thimerosal-containing hepatitis B vaccine. The Advisory Committee on Immunization Practices (ACIP) in 1991 recommended the following hepatitis B vaccination schedule for infants—first dose (birth to 2 months of age), second dose (1 to 4 months of age), and third dose (6 to 18 months of age) [18].

An additional strength of this study was that the controls subjects were observed for a long enough period of time to ensure that they were likely to remain controls. It was *a priori* established that controls should be continuously HMO-enrolled from birth, until mean age of initial premature puberty diagnosis plus the standard deviation, which was 5.59 years. As revealed in Table 5, by reducing the length of continuous HMO-enrollment from birth until the mean age of initial premature puberty diagnosis, which was 2.94 years, the overall effects observed were significantly reduced. Despite the strength of this study being able to follow the controls who were continuously HMO-enrolled from birth until 5.59 years-old, it was not possible for us to continue to follow controls beyond 5.59 years because the publically available VSD data ended at the end of 2000. It is recommended that in future studies controls be followed for long periods, to see what potential effects it may have on the phenomena observed in this study.

This study may have another limitation in that potentially unknown biases or cofounders could have contributed to the association observed between Hg exposure from Thimerosal-containing hepatitis B vaccine and the risk of diagnosed premature puberty. This would seem unlikely because previous studies have examined the potential dose-dependent relationship between Hg exposure from Thimerosal-containing hepatitis B vaccines and diagnosed outcomes, such as febrile seizures, failure to thrive, and cerebral degenerations [19]. These conditions were chosen because they were hypothesized to not be plausibly related, biologically, to Hg exposure from Thimerosal-containing hepatitis B vaccines. It was observed that there were no increasing dose-response relationships between the increasing levels of exposure to Hg from the Thimerosal-preserved hepatitis B vaccine and the risk of the children having any of these non-biologically plausible outcomes (febrile seizures, failure to thrive, and cerebral degeneration). In addition, unknown biases or confounders accounting for the results observed in this study are further minimized because, as formerly described, a previous ecological cohort study, using different epidemiological methods, observed a similar dose-response association between the risk of a premature puberty diagnosis and exposure to vaccines containing Hg from the Thimerosal preservative [10].

A further potential limitation of this study was that the risk of being diagnosed with premature puberty is influenced by socioeconomic-associated environmental or ethnic/racial factors. It is possible that study subjects receiving additional Hg exposure from Thimerosal-containing hepatitis B vaccines may have other environmental or ethnic/racial factors, but it is important to consider that all study subjects examined, regardless of their socioeconomic or ethnic/racial backgrounds, had enough financial means to be continuously enrolled at a participating HMO from their date of birth, for a substantial length of time. It would be worthwhile in future studies to consider these factors to see, what effects, if any, they have on the phenomena observed in this study.

It is also a potential limitation of this study that statistical chance may account for the phenomena observed. It would seem unlikely, given the significance and consistency of the findings observed. This is further supported by the fact that the results observed were apparently gender and dose-dependent. It would be worthwhile in future studies to see if they confirm and extend the results observed in this study.

A still further potential limitation of this study was that misdiagnoses may have occurred or inappropriate recording or classification of some vaccine exposure may not have occurred among some of the individuals examined. It was assumed that while these errors may have occurred, they should have occurred at a similar rate among the cases and the controls examined. In addition, such errors would reduce the statistical power of this study to observe true potential exposure–outcome associations, by biasing the results toward the null hypothesis, because individuals would be placed in the wrong outcome/exposure categories.

An additional limitation of this research possibly occurred because it examined medically diagnosed premature puberty, and as such, no differential diagnosis of the etiology was undertaken. It is possible that some of the children diagnosed with premature puberty, examined in this study, may have had, for example, central nervous system tumors, congenital adrenal hyperplasia, teratomas, pituitary tumors or adrenal/ovarian tumors, which may have other known causes, besides Hg. The result in the present study may underestimate the true relationship between Thimerosal exposure and the risk of being diagnosed with premature puberty. Future studies should further examine this potential phenomenon.

There are also many potential additional causes of Hg exposure, beyond the source evaluated in this study, and as such, this might represent a potential limitation of this study. It is most probable that the children examined in this study received an additional Hg exposure from other Thimerosal-preserved immunizations, formula, breast feeding, amalgams, dietary seafood, the environment, etc. It is believed that the presence of these other sources of Hg exposure, among the children examined in this study, would actually tend to bias the results observed in this study towards the null hypothesis. This may have occurred because individuals may have been misclassified as to their true total Hg exposure level, since only one source of Hg was examined, i.e., hepatitis B vaccines containing Hg from Thimerosal. Unfortunately, the database of the VSD does not provide records regarding other sources of Hg exposure, and while it does contain information about other vaccine exposures children received, the CDC, in preparing our VSD datasets, limited us to being able to study only on vaccine type, at a time. It would be worthwhile in future studies to examine, what effects, if any, these other sources of Hg have on the potential relationship between increasing Hg exposure and increasing risk of being diagnosed with premature puberty. In addition, this study did not examine whether other covariates, e.g., weight at birth, race, additional diagnoses, etc. that could modify the observed effects. These covariates should be further explored in future studies. Furthermore, this study did not examine any other potential exposures to endocrine disrupters, such as aluminum or glyphosates [20], or any potential synergistic relationship between them and Thimerosal. Aluminum would be particularly important to examine since it is added to some vaccines as an adjuvant [21], and because recent evidence shows that when Hg is co-exposed with aluminum there can be worsened effects. For example, Alexandrov et al. [21], in 2018, studied Hg effects alone and in combination with aluminum in human neuronal cells, in the presence of astroglial cells. Exposure to 20 nM of mercury sulfate alone induced an inflammatory response that was approximately two-fold above the background. When Hg was co-exposed with aluminum, the inflammatory response was approximately four-fold above the background.

Finally, how generalizable are the results (found from the VSD database) to the whole populace of the US, is of concern as a potential limitation of this study. The CDC, in a recent study, considered this possible limitation of this specific database [22]. They concluded that the VSD database is indeed large enough to contain a diverse enough population, to make sure there is adequate representation of the whole US population.

## 6. Conclusions

In conclusion, this longitudinal case-control study provides additional epidemiological evidence, based upon examination of prospectively collected medical records, of a significant overall and dose-dependent association between the increasing organic Hg exposure from Thimerosal-containing hepatitis B vaccines, administered within the first six months of life and the long-term risk of the child being diagnosed with premature puberty. The phenomena support the notion that effects from exposure to organic Hg from Thimerosal-containing hepatitis B vaccines are mediated by the timing of exposure and potentially by the gender of the recipient. The overall results of this study are supported by previous biological plausibility evidence implicating Hg as an endocrine disruptor, and support that the mercurial compound Thimerosal is also an endocrine disruptor.

The findings of this research are relevant in preventive medicine today because Thimerosal is still used as a preservative in some vaccines in the US and abroad (particularly, in the developing countries). For instance, in the US, over half of all influenza-vaccine doses, more than 75 million per year, still use Thimerosal as a preservative. The influenza vaccine is recommended for pregnant women and also infants and children (twice in the first year for infants). Thimerosal is also still used in the US in the meningococcal vaccine, as well as the tetanus-toxoid vaccine. Thimerosal is still used in many childhood vaccines, in the developing world. In addition, tetanus and influenza vaccines, both of which potentially contain Thimerosal, are recommended for pregnant women in the developing world [7].

Two more important points are worth mentioning. First, there are serious potential late sequelae of premature puberty for females. These include, but are not limited to, shortened height, cardiovascular disease, mental health issues, and obesity [10,23]. Second, Thimerosal has failed testing for efficacy, indicating that it is ineffective as a preservative, especially with *Staphylococcus aureus* [24].

The results of this current study imply that if Hg were no longer added to the immunizations, it could significantly impact the rates of premature puberty among children. Vaccines that are routinely administered to children, are an important public health instrument to reduce infectious disease associated with morbidity/mortality [25], however, in light of the findings of this study and other recent studies, the use of Thimerosal in vaccines needs to be eliminated.

## Figures and Tables

**Table 1:** This table summarizes the Hg exposure from Thimerosal-preserved HepB immunizations administered among cases with a premature puberty diagnosis compared to the control subjects ^1^, within the database of the VSD.

Group Examined	Number of Cases Diagnosed with Premature Puberty (%)	Number of Controls without a Premature Puberty Diagnosis (%)	Odds Ratio (95% CI)	*p*-Value
*Group I*				
12.5 µg Hg within 1st month	255 (52.47)	20,582 (37.97)		
			1.803 (1.508–2.156)	<0.00001
0 µg Hg within 1st month	231 (47.53)	33,617 (62.03)		
*Group II*				
25 µg Hg within first 2 months	254 (52.59)	20,604 (38.56)		
			1.768 (1.477–2.115)	<0.00001
0 µg Hg within first 2 months	229 (47.41)	32,835 (61.44)		
*Group III*				
37.5 µg Hg within first 6 months	56 (81.16)	3914 (67.27)		
			2.0955 (1.143–3.841)	<0.05
0 µg Hg within first 6 months	13 (18.84)	1904 (32.73)		

^1^ The control subjects were enrolled continuously from their birth, for a minimum of 5.59 years (the mean age of their initial premature puberty diagnosis plus the SD of mean age of their initial premature puberty diagnosis). Hg = mercury, µg = microgram, SD = standard deviation.

**Table 2:** This study summarizes the exposure to Hg from Thimerosal-preserved HepB immunizations administered among male cases with a premature puberty diagnosis compared to the male control subjects ^1^, within the database of the VSD.

Group Examined	Number of Male Cases Diagnosed with Premature Puberty (%)	Number of Male Controls without a Premature Puberty Diagnosis (%)	Odds Ratio (95% CI)	*p*-Value
*Group IV*				
12.5 µg Hg within 1st month	10 (35.71)	10,584 (37.81)		
			0.914 (0.422–1.98)	>0.99
0 µg Hg within 1st month	18 (64.29)	17,405 (62.19)		
				
*Group V*				
25 µg Hg within first 2 months	9 (33.33)	10,594 (38.40)		
			0.802 (0.360–1.786)	>0.65
0 µg Hg within first 2 months	18 (66.67)	16,991 (61.60)		
				
*Group VI*				
37.5 µg Hg within first 6 months	3 (75.00)	1979 (67.06)		
			1.474 (0.153–14.184)	>0.99
0 µg Hg within first 6 months	1 (25.00)	972 (32.94)		

^1^ The control subjects were enrolled continuously from their birth, for a minimum of 5.59 years (the mean age of their initial premature puberty diagnosis plus the SD of mean age of their initial premature puberty diagnosis). Hg = mercury, µg = microgram, SD = standard deviation.

**Table 3:** This table summarizes the organic Hg exposure from the hepatitis B vaccine preserved with Thimerosal in the female cases with a premature puberty diagnosis compared to the female controls ^1^, within the database of the VSD.

Group Examined	Number of Female Cases Diagnosed with Premature Puberty (%)	Number of Female Controls without a Premature Puberty Diagnosis (%)	Odds Ratio (95% CI)	*p*-Value
*Group VII*				
12.5 µg Hg within 1st month	245 (53.49)	9997 (38.14)		
			1.865 (1.55–2.245)	<0.00001
0 µg Hg within 1st month	213 (46.51)	16,212 (61.86)		
*Group VIII*				
25 µg Hg within first 2 months	245 (53.73)	10,009 (38.72)		
			1.838 (1.526–2.213)	<0.00001
0 µg Hg within first 2 months	211 (46.27)	15,844 (61.28)		
*Group IX*				
37.5 µg Hg within first 6 months	53 (81.54)	1935 (67.49)		
			2.127 (1.131–4.000)	<0.05
0 µg Hg within first 6 months	12 (18.46)	932 (32.51)		

^1^ The control subjects were enrolled continuously from their birth, for a minimum of 5.59 years (the mean age of their initial premature puberty diagnosis plus the SD of mean age of their initial premature puberty diagnosis). Hg = mercury, µg = microgram, SD = standard deviation.

**Table 4:** This table summarizes the logistic-regression results of the effect of exposure to organic Hg in Thimerosal-preserved hepatitis B vaccines, used within the first six months after birth, for female cases with a premature puberty diagnosis compared to the female controls ^1^.

Group Examined (ICD-9 Code)	Reference Dose 0 µg Hg (%)	12.5 µg Hg (%)	25 µg Hg (%)	37.5 µg Hg (%)	Odds Ratio Per µg Hg (95% CI) [*p*-Value]
Female Premature Puberty Cases (259.1)	12 (2.62)	2 (0.44)	391 (85.37)	53 (11.57)	
					1.0281 (1.0104–1.0462) [<0.005]
Female Controls	932 (3.57)	356 (1.36)	22,896 (87.66)	1935 (7.41)	

^1^ The control subjects were enrolled continuously from their birth, for a minimum of 5.59 years (the mean age of their initial premature puberty diagnosis plus the SD of mean age of their initial premature puberty diagnosis). Hg = mercury, µg = microgram, SD = standard deviation.

**Table 5:** This table summarizes the exposure to Hg from Thimerosal-preserved HepB vaccines among the cases with a premature puberty diagnosis, compared to control subjects, with a reduced length of follow-up ^1^, within the VSD database.

Group Examined	Number of Cases Diagnosed with Premature Puberty (%)	Number of Controls without a Premature Puberty Diagnosis (%)	Odds Ratio (95% CI)	*p*-Value
*Group I*				
12.5 µg Hg within 1st month	255 (52.47)	55,606 (44.79)		
			1.361 (1.138–1.626)	<0.001
0 µg Hg within 1st month	231 (47.53)	68,539 (55.21)		
*Group II*				
25 µg Hg within first 2 months	254 (52.59)	55,615 (45.30)		
			1.34 (1.120–1.602)	<0.01
0 µg Hg within first 2 months	229 (47.41)	67,163 (54.70)		
*Group III*				
37.5 µg Hg within first 6 months	56 (81.16)	11,786 (72.65)		
			1.622 (0.886–2.969)	>0.10
0 µg Hg within first 6 months	13 (18.84)	4438 (27.35)		

^1^ The controls were continuously enrolled from their date of birth for a minimum of 2.94 years (mean age of the initial premature puberty diagnosis. Hg = mercury, µg = microgram.
